# High plasma resistin associates with severe acute kidney injury in Puumala hantavirus infection

**DOI:** 10.1371/journal.pone.0208017

**Published:** 2018-12-05

**Authors:** Paula S. Mantula, Tuula K. Outinen, Pia Jaatinen, Mari Hämäläinen, Heini Huhtala, Ilkka H. Pörsti, Antti Vaheri, Jukka T. Mustonen, Satu M. Mäkelä

**Affiliations:** 1 Tampere University Hospital, Department of Internal Medicine, Tampere, Finland; 2 Faculty of Medicine and Life Sciences, University of Tampere, Tampere, Finland; 3 Division of Intermal Medicine, Seinäjoki Central Hospital, Seinäjoki, Finland; 4 The Immunopharmacology Group, Faculty of Medicine and Life Sciences, University of Tampere and Tampere University Hospital, Tampere, Finland; 5 Faculty of Social Sciences, University of Tampere, Tampere, Finland; 6 Department of Virology, Medicum, University of Helsinki, Helsinki, Finland; Robert Bosch Krankenhaus, GERMANY

## Abstract

**Background:**

Puumala hantavirus (PUUV) infected patients typically suffer from acute kidney injury (AKI). Adipokines have inflammation modulating functions in acute diseases including AKI. We examined plasma levels of three adipokines (resistin, leptin, and adiponectin) in acute PUUV infection and their associations with disease severity.

**Methods:**

This study included 79 patients hospitalized due to acute PUUV infection. Plasma resistin, leptin, adiponectin, as well as IL-6 and CRP, were measured at the acute phase, recovery phase and one year after hospitalization.

**Results:**

Plasma resistin levels were significantly higher in the acute phase compared to the recovery phase and one year after (median resistin 28 pg/mL (11–107) vs. 17 pg/mL (7–36) vs. 14 pg/mL (7–31), p<0.001). Maximum resistin concentration correlated with maximum plasma creatinine levels (r = 0.63; p<0.001). The higher the amount of albuminuria in the urine dipstick test (0–1+, 2+ or 3+) at admission, the higher the median of maximum resistin (24.7 pg/mL, 25.4 pg/mL and 39.6 pg/mL, respectively, p = 0.002). High resistin was also an independent risk factor for severe AKI (creatinine ≥353.6μmol/L) (OR 1.08, 95% CI 1.02–1.14). Neither plasma leptin nor adiponectin level had any correlation with creatinine concentration or the amount of albuminuria.

**Conclusions:**

Plasma resistin independently associates with the severity of AKI in acute PUUV infection. The association of resistin with the amount of albuminuria suggests that the level of plasma resistin is not only influenced by renal clearance but could have some role in the pathogenesis of AKI during PUUV infection.

## Introduction

Puumala virus (PUUV) belongs to the family of hantaviruses and it is spread by the bank vole (*Myodes glareolus*). In humans, PUUV causes an illness known as Nephropatia Epidemica (NE) [1]. In Finland, thousands of serologically confirmed diagnoses are made annually and the number of infected humans parallels the population of the bank vole. The disease is transmitted by inhaling dust contaminated by PUUV-infected bank vole feces or urine [2].

The characteristic manifestation of PUUV infection is a mild form of hemorrhagic fever with renal syndrome (HFRS) [3, 4]. Although many of the diagnosed patients need hospital treatment, the majority of the infections are asymptomatic or cause only mild and transient symptoms and thus remain undiagnosed [5]. In a Finnish cohort, 83% of hospital-treated patients had acute kidney injury (AKI) [6]. This syndrome has a good prognosis: kidney function returns to normal in practically all patients by supportive therapy and the mortality is low, around 0,1% [6, 7]. Several long-term nephrological, cardiovascular and endocrinological consequences have, however, been described after PUUV infection [5].

Proteinuria and hematuria are typical urinary findings in the early phase of the infection. Proteinuria is often of nephrotic range (>3g/day) and resolves rapidly. It might be a sign of change in the barrier function of the glomerular vasculature [7]. The histological finding is acute tubulointerstitial nephritis while glomerular changes are minimal [8]. Immunohistochemical studies reveal abnormalities preferentially in the tubuli and peritubular areas, where infiltrating cells consist of plasma cells, monocytes/macrophages, eosinophils and neutrophils. At the same site, the expression of tumor necrosis factor (TNF)-α as well as endothelial adhesion molecules intercellular adhesion molecule (ICAM)-1 and vascular cell adhesion molecule (VCAM)-1 are seen, as a sign of endothelial cell activation [9].

Factors affecting the severity of PUUV infection remain mostly unclear, but host genetic factors have an influence [10]. Inflammatory markers have been shown to increase during acute PUUV infection, including plasma and urinary interleukins (ILs) IL-6, IL-1β, IL1 receptor antagonist, TNF-α, and soluble urokinase-type plasminogen activator receptor, as well as plasma pentraxin-3 and urinary gelatinase-associated lipocalin (NGAL) [11–15]. Particularly urinary IL-6 and urinary NGAL [12, 16] have been found to associate with the severity of AKI. The association of widely used C-reactive protein (CRP) with severe AKI in PUUV infection is less clear [17] [12]. We have previously found that the amount of proteinuria and hematuria in dipstick sample at the acute phase is associated with the severity of AKI in NE patients [18, 19]. The peak of proteinuria seems to precede the most severe phase of AKI [18].

Adipokines—also called adipocytokines—are bioactive molecules first found to be secreted by adipose tissue, and to regulate appetite and energy metabolism. More recently, adipokines have been discovered to be produced by many other cell types, particularly by inflammatory cells, and to regulate inflammatory responses [20]. Interestingly, plasma resistin changes have been reported in acute infections [21, 22] and AKI [23].

In the present study, our aim was to determine the adipokines adiponectin, leptin and resistin in PUUV infection, and examine their associations with the severity of acute PUUV infection and the concomitant AKI.

## Materials and methods

The study cohort originally consisted of 86 consecutive patients with acute, serologically confirmed PUUV infection treated at the Tampere University Hospital in Finland, during Jan 2005-Nov 2014. Plasma samples for adipokine measurements were available in 79 patients and these patients comprised the final study cohort. Detailed medical history was obtained and physical examination was performed during the acute phase of the disease. All patients provided a written informed consent and the study was approved by the Ethics Committee of Tampere University Hospital (R04180, R09206).

Acute PUUV infection was confirmed from a single serum sample by detecting the typical granular staining pattern in immunofluorescence assay [24], and/or low avidity of IgG antibodies to PUUV [25], and/or by detecting PUUV IgM antibodies by an ‘in-house’ enzyme-linked immunosorbent assay (ELISA) based on a recombinant antigen [26].

Plasma samples for the measurement of resistin, leptin and adiponectin concentrations as well as CRP and IL-6 levels were collected between 7:30–8:30 am, a median of 2 (1–5) times during the acute phase. The highest or the lowest values (as appropriate) of the various variables measured during the hospital stay were designated as the maximum or minimum values. The follow-up samples were obtained a median of 15 (range 7–21) days after discharge from hospital in 74 patients, and at one year after hospitalization in 67 patients. Plasma resistin, leptin, adiponectin, CRP and IL-6 concentrations were measured by an enzyme-linked immunosorbent assay (ELISA) using reagents from R&D Systems Europe Ltd, Abingdon, UK (resistin, leptin, adiponectin and CRP) and from eBioscience Inc, San Diego, CA, USA (IL-6). The detection limit and interassay coefficient of variation were 15.6 pg/mL and 8.5% for resistin, 15.6 pg/mL and 5.3% for leptin, 15.6 pg/mL and 6.0% for adiponectin, 3.9 pg/mL and 5.7% for CRP, and 0.39 pg/mL and 4.8% for IL-6. For adiponectin, the test detects total adiponectin.

Plasma creatinine was measured daily during hospitalization, median 5 (2–13) measurements per patient, by a Cobas Integra (F. Hoffman- La Roche Ltd., Basel, Switzerland). A urine dipstick test was performed on admission to hospital. The urine dipstick analysis was performed by automated tests based on refractometry (Siemens Clinitec Atlas or Advantus). The sensitivity of these semi-quantitative dipstick tests to urine albumin (1+) ranges 0.15–0.3 g/l. The dipstick result 2+ indicates >1 g/l albumin, and the result 3+ >3 g/l albumin. Assay for hematuria detects heme pseudoperoxidase activity and therefore it detects red cell casts and dysmorphic red cells also. The sensitivity of the assay is about 10 x 10^6^ cells/L (about 3–5 cells by high power field).

Severe AKI was defined as maximum plasma creatinine level ≥353.6 μmol/L during hospitalization (stage 3, according to KDIGO definition) [22]. The amount of hourly urine output was not recorded. Here, shock is defined by a fall in systolic blood pressure under 90 mmHg with clinical symptoms of shock. Body mass index (BMI) was calculated as the ratio of weight (kg) to squared height (m^2^).

## Statistical analyses

The data is presented as medians and ranges for continuous variables and numbers and percentages for categorical variables. Groups were compared using the Mann–Whitney U test or the Kruskal-Wallis test, as appropriate. Correlations were calculated by the Spearman rank correlation test. Related samples were compared using the Wilcoxon signed rank test or the Friedman test, as appropriate. A receiver operating charasteristic (ROC) curves were drawn in order to evaluate which of the maximum levels of various inflammatory markers could act as the best indicator of severe AKI (plasma creatinine ≥353.6 μmol/L). A logistic regression analysis was performed with age, sex, BMI, dipstick-albuminuria class (0/1+,2+ and 3+) and maximum plasma resistin level as independent factors to examine the association of these factors with severe AKI. Adjusted odds ratios (OR) and their 95% confidence intervals (95% CI) are given. The goodness of fit was assessed with Hosmer Lemeshow statistic. All tests were two-sided, and p-values <0.05 were considered statistically significant. The SPSS statistical software package (IBM SPSS Statistics version 23.0 Armonk, NY, USA) was used for all analyses.

## Results

The clinical characteristics and laboratory findings of the patients are listed in Table 1. The median length of hospital stay was 6 days. The median age was 41 years (range 21–74) and 48 (61%) of the patients were males. The following diagnoses had been made before the acute PUUV infection in 24 (30%) patients: hypertension (n = 7), asthma/chronic obstructive pulmonary disease (n = 4), gastritis/reflux disease (n = 4), rheumatoid arthritis (n = 3), coronary artery disease (n = 2), type 2 diabetes (n = 2), type 1 diabetes (n = 1), and transient ischemic attack (n = 1). Some of the patients had more than one disease, but none had a known kidney disease or chronic renal insufficiency.

**Table 1:** Clinical and laboratory findings in 79 patients hospitalized due to acute Puumala hantavirus infection.

Finding	Median	Range
Age (years)	41	21–74
Body mass index, n = 72 (kg/m^2^)	26	18–37
Duration of fever (days)	8	4–15
Length of hospital stay (days)	6	2–14
Systolic blood pressure on admission (mmHg)	126	72–182
Change in body weight during hospital stay (kg)	2	0–11
Plasma creatinine max (μmol/L)	186	51–1499
Hematocrit max	0.44	0.33–0.60
Platelets min (x10^9^/L)	52	5–150
Plasma sodium min (mmol/L)	130	109–139
Plasma potassium max (mmol/L)	4.2	3.3–5.3
Plasma albumin min (g/L)	25	18–34
Leukocytes max (x10^9^/L)	10.8	4.2–45.0
IL-6 max (ρg/mL)	11.8	1.6–66.6
CRP max (ρg/mL)	57	8–199

Min, minimum value during hospital stay; max, maximum value during hospital stay; IL-6, Interleukin-6; CRP, C-reactive protein

The elevation of plasma creatinine level above 100 μmol/L was detected in 56 (70%) patients. Severe AKI (creatinine ≥353.6 μmol/L) occurred in 25 (32%) patients. One patient needed transient dialysis treatment and two patients suffered from a clinical shock on admission to hospital. All of the patients recovered. The median of the maximum plasma creatinine at the acute phase was 186 μmol/L (range 51–1499) (Table 1). At the recovery phase (15 days after hospitalization) and one year after the acute infection, the median of creatinine was 78 μmol/L (range 55–184) and 71 μmol/L (range 53–123) respectively.

**Fig 1:**
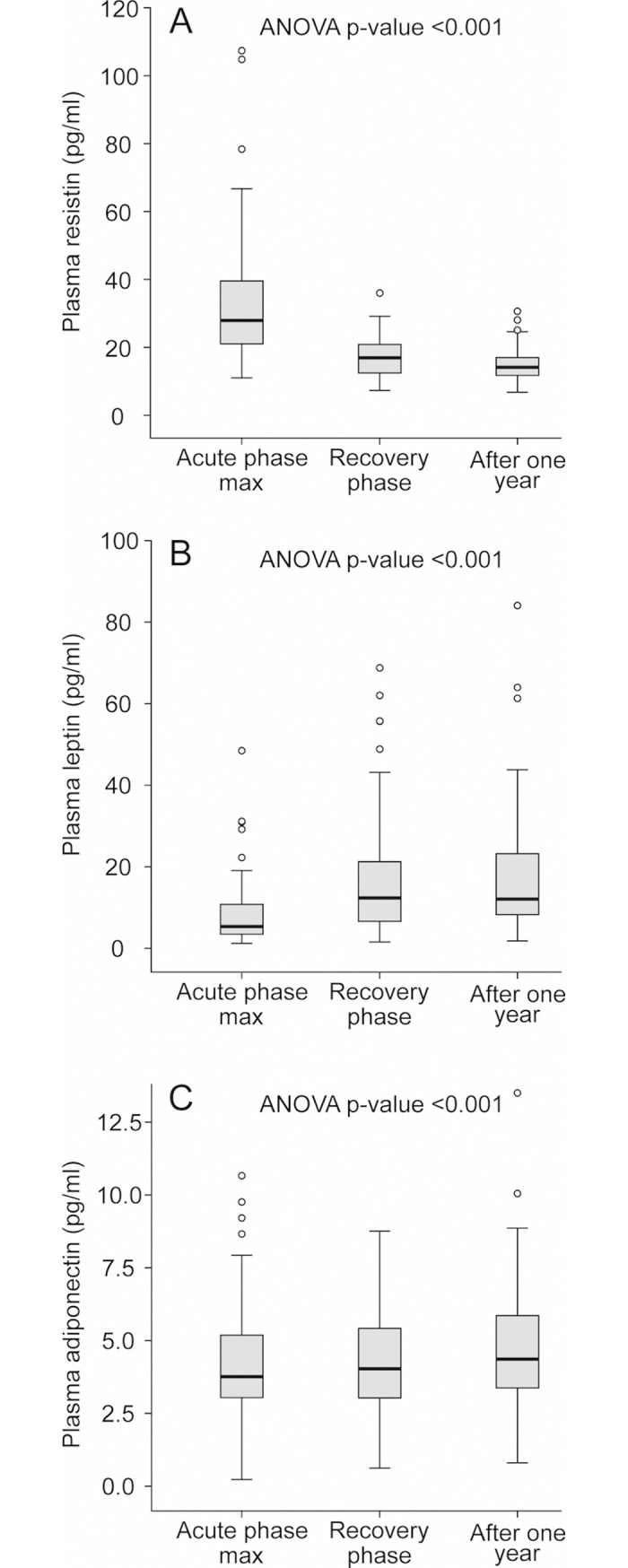
**Plasma resistin (A), leptin (B), and adiponectin (C) levels during acute Puumala hantavirus (PUUV) infection, in the recovery phase, and one year after the hospitalization.** Median (thick line inside box), 25th-75th percentiles (box), range (whiskers), and outliers (○).

The changes in plasma adipokine, IL-6 and CRP levels in the acute phase of PUUV infection, compared to the recovery phase and one year after hospitalization, are presented in Table 2. and Fig 1. The median time to the first adipokine measurement from the onset of fever was 7 days (range 3–14). The resistin levels (Fig 1A) were significantly higher and leptin levels (Fig 1B) significantly lower in the acute phase, compared to those measured in the recovery phase or one year after the acute illness. There was also a slight but statistically significant decrease in adiponectin level in the acute phase compared to the recovery phase or one year after the acute illness (Fig 1C).

**Table 2:** The levels of plasma adipokines (resistin, leptin, adiponectin), and other markers of inflammation (leukocytes, plasma IL-6, CRP) in the acute phase of Puumala hantavirus infection compared to the recovery phase and one year after the infection.

	Acute phase* (n = 79)			Recovery phase (n = 74)		After 1 year (n = 67)		p-value
	median		range	median	range	median	range	
Resistin (ρg/mL)	28		11–107	17	7–36	14	7–31	<0.001
Leptin (ρg/mL)	5.3		1.2–48.4	12.2	1.6–68.7	12.1	1.8–84.0	<0.001
Adiponectin (ρg/mL)	3.76		0.23–10.66	4.07	0.62–10.25	4.36	0.80–13.49	<0.001
Leukocytes (x10^9^/L)	10.8		4.2–44.4	7.6	3.7–14.5	6.7	3.7–11.4	<0.001
IL-6 (ρg/mL)	11.8		1.7–66.6	1.2	0.4–12.5	0.9	0.4–15.9	<0.001
CRP (ρg/mL)	57.3		8.4–198.5	1.7	0.2–36.0	1.3	0.1–13.2	<0.001

IL-6, interleukin-6; CRP, C-reactive protein. P-value stands for the differences between the three phases.

*Acute phase values are maximum (resistin, leukocytes, IL-6, CRP) or minimum (leptin, adiponectin)

The correlations of maximum plasma resistin concentration, minimum plasma adiponectin and minimum plasma leptin with clinical and laboratory variables in the acute phase of PUUV infection are shown in Table 3. The high resistin level correlated with higher maximum plasma creatinine concentration. The median of maximum resistin was significantly higher in patients with creatinine level >100 μmol/L compared to those with maximum creatinine ≤ 100 μmol/L (resistin 32 ρg/mL, range 11–107 vs. 21 ρg/mL, range 11–42, p<0.001). The maximum resistin values also correlated with many other variables reflecting disease severity (Table 3). Interestingly, the highest measured resistin value of this cohort (107 pg/mL) was detected in one of the two patients with a clinical shock. The changes in the other two adipokines, minimum adiponectin and minimum leptin didn’t have any clear correlations with the clinical disease severity markers in PUUV infection (Table 3).

**Table 3:** Correlations of plasma resistin concentration with clinical and laboratory markers of disease severity in acute Puumala hantavirus infection.

	Resistin maximum, r	p-value	Adiponectin minimum, r	p-value	Leptin minimum, r	p-value
Duration of hospital stay	0.507	<0.001	0.004	0.970	0.142	0.213
Systolic blood pressure on admission	-0.257	0.022	-0.240	0.033	-0.103	0.368
Change in body weight during hospital stay	0.433	<0.001	-0.017	0.888	0.002	0.988
Creatinine max	0.633	<0.001	0.029	0.802	-0.044	0.700
Platelets min	-0.254	0.024	-0.085	0.456	0.106	0.352
Sodium min	-0.368	0.001	-0.015	0.895	0.241	0.032
Potassium max	0.369	0.001	-0.004	0.971	0.038	0.742
Leukocytes max	0.520	<0.001	0.060	0.600	-0.078	0.495
IL-6 max	0.329	0.003	-0.270	0.016	-0.160	0.160
CRP max	-0.071	0.536	-0.457	<0.001	-0.034	0.763

max, maximum; min, minimum; CRP,C-reactive protein; IL-6, interleukin-6

The maximum plasma creatinine level measured during the hospital stay, didn’t have any correlation with BMI (r = 0.145;p = 0.223). The minimum plasma leptin concentration had a weak correlation with BMI (r = 0.350; p = 0.003) but not with maximum creatinine values (Table 3).

Proteinuria on hospital admission (defined as urine albumin dipstick test ≥2+) was detected in 54 (68%) patients. Hematuria (≥2+ in urine dipstick test) was detected in 34 (43%) of the patients. When analyzing the amount of proteinuria categorized by dipstick albuminuria 0/1+, 2+ or 3+ at the acute phase, the maximum resistin level was significantly higher in patients with albuminuria 3+ than in patients with 0/1+ or 2+ albuminuria (Table 4). The other adipokines studied were not associated with dipstick-albuminuria. When combining albuminuria and hematuria findings in the urine dipstick test (0–2+, 3–4+ or 5–6+), the higher the combined positive result, the higher was the maximum plasma resistin concentration (Table 4).

**Table 4:** Plasma resistin levels in different categories of urine dipstick albuminuria and hematuria in 79 patients with Puumala hantavirus infection.

Urine dipstick albumin	Resistin max median (pg/mL)	range	
0–1+ (n = 25)	24.7	11.6–90.7	
2+ (n = 24)	25.4	11.9–80.4	p = 0.002
3+ (n = 30)	39.6	11.1–107.3	
**Urine dipstick albumin+erythrocytes**			
0–2+ (n = 26)	22.2	11.6–90.7	
3–4+ (n = 35)	27.1	11.1–80.4	p<0.001
5–6+ (n = 18)	42.7	16.8–107.3	

p-value stands for the differences between the three groups

**Fig 2:**
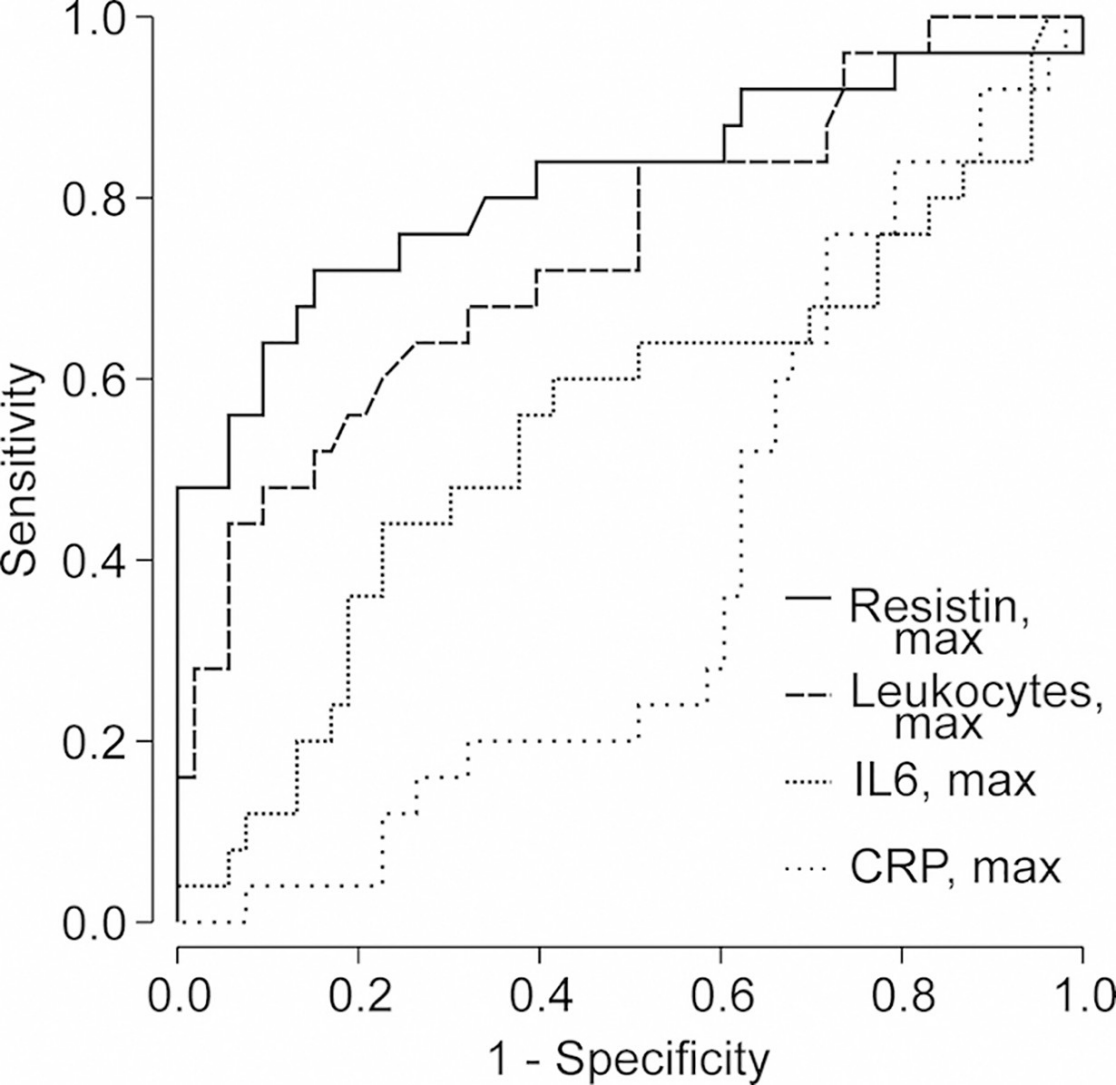
Receiver operating characteristic (ROC) curves for plasma resistin and different plasma inflammatory markers (maximum C-reactive protein, maximum interleukin-6, and maximum leukocyte count) in predicting severe acute kidney injury (plasma creatinine ≥353.6 μmol/L) during acute Puumala hantavirus infection.

We generated receiver operating curves (ROC) for the maximum plasma resistin, IL-6 and CRP levels, as well as for the maximum leukocyte count, in the acute phase to discriminate upcoming severe AKI (creatinine ≥353.6 μmol/L) from non-severe AKI (Fig 2). Resistin was a stronger discriminator with area under ROC curve (AUROC) of 0.82 (95%CI 0.70–0.91, p<0.001) when compared with leukocyte count (AUROC 0.74, 95% CI 0.62–0.87; p = 0.001). IL-6 or CRP did not discriminate severe AKI from non-severe AKI (AUROC 0.55, 95% CI 0.40–0.70; p = 0.493 and AUROC 0.39, 95% CI 0.26–0.51; p = 0.105, respectively).

We performed a logistic regression analysis to evaluate the associations of age, gender, BMI, dipstick albuminuria and plasma resistin level with severe AKI (Table 5). In a univariate model, male gender, dipstick albuminuria of 3+, and maximum resistin level were all associated with severe AKI. In the multivariable analysis only male gender and maximum plasma resistin level were found to be independent risk factors for severe AKI.

**Table 5:** Multivariate logistic regression analysis of risk factors for severe AKI (plasma creatinine ≥353.6 μmol/L) among 79 hospitalized patients with acute Puumala hantavirus infection.

			Univariate		Multivariable	
	No severe AKI n = 54	Severe AKI n = 25	OR	95% CI	OR	95% CI
	Median (range)	Median (range)				
Age (years)	42 (22−67)	39 (21−74)	0.99	0.96−1.03	1.00	0.95−1.06
Sex (n)						
Female	28	3	1		1	
Male	26	22	7.90	2.11−29.54	6.73	1.16−38.90
BMI (kg/m^2^) n = 72	25.8 (18.5−37.0)	26.2 (22.6−32.3)	1.04	0.91−1.18	1.00	0.81−1.23
Albumin dipstick (n)						
0–1+	22	3	1		1	
2+	20	4	1.47	0.29−7.37	0.81	0.10−6.70
3+	12	18	11.00	2.69−45.06	4.61	0.76−28.08
Resistin max (ρg/mL)	25.0 (11.6−52.6)	45.0 (11.0−107.3)	1.10	1.05−1.154	1.08	1.02−1.14

Max = maximum, min = minimum, IL-6 = interleukin-6, CRP = C-reactive protein.

Multivariable analysis Hosmer—Lemeshow test p = 0.591

## Discussion

The present study shows that plasma resistin concentrations are elevated in the acute phase of PUUV hantavirus infection. High resistin levels correlated with the severity of AKI, as well as with several other markers reflecting the severity of the disease. High plasma resistin levels during the acute infection also associated with pronounced albuminuria and hematuria detected with urine dipstick test at hospital admission. Plasma resistin showed an independent influence on severe AKI.

Resistin was first described in 2001 when it was found to be produced by adipose tissue and promote insulin resistance in mice [27]. Further studies showed that in humans, resistin is mainly produced by leukocytes, especially macrophages, and its contribution to the development of insulin resistance remains unclear [28, 29]. The role of resistin as an inflammatory factor started to be unraveled by the early finding that injection of bacterial lipopolysaccharide (LPS) into healthy volunteers caused a rise in circulating resistin levels [30]. Specific receptors for resistin have not been identified, but it belongs to the endogenous ligands of the inflammation triggering toll-like receptor 4 (TLR-4) [31]. Accordingly, resistin is associated with an array of inflammatory diseases including sepsis, inflammatory bowel disease, arthritis and astma [32, 33]. We have previously found resistin as a contributing factor and biomarker involved in osteoarthritis [34], rheumatoid arthritis [35], inflammatory lung diseases [36, 37] and ischemia-reperfusion syndrome associated with cardiac surgery [38]. Interestingly, resistin seems also to be produced by tumors / tumor associated macrophages. For instance, resistin was recently reported as a predictive factor for the recurrence and long-term prognosis in renal cell cancer [39]. Furthermore, plasma resistin levels have been found to be significantly higher in patients with septic shock and AKI, when compared with patients with septic shock without AKI, and it was found to modulate the inflammatory response in those patients [23].

There are a few studies investigating the significance of resistin in other acute virus infections. Plasma resistin was elevated in the early phase of acute Dengue fever, but associations with kidney function or other disease severity markers were not reported [22]. In patients with Crimean-Congo hemorrhagic fever, caused by tick-borne virus, plasma resistin was elevated and higher concentrations associated with severe disease, defined by the manifestations of bleeding. Resistin had a negative correlation with platelet count, but it did not have a correlation with plasma creatinine. The kidney function of these patients was reported to be almost normal and AKI was not addressed [40].

The plasma resistin elevation in PUUV infection could be a sign of inflammation as previously shown in sepsis [21]. Plasma resistin is elevated in patients with septic shock [41, 42]. In the present study, only two patients suffered from clinical shock, one of them having the highest plasma resistin concentration of this cohort. Furthermore, high resistin level correlated with low systolic blood pressure at admission. The clinical shock syndrome in PUUV infection is related to an increased vascular permeability, which probably has a role also in the pathogenesis of the nephrotic-range proteinuria [7].

In adult intensive care unit (ICU) patients, resistin was superior to CRP in distinguishing sepsis from systemic inflammatory response (SIRS) due to trauma without infection, and the level of resistin was significantly higher in sepsis compared to trauma related SIRS [43]. In ICU patients, high resistin was shown to associate with worse long-time-survival in non-septic patients, maybe as a sign of considerable harm of excessive inflammatory response [44]. We have previously reported that plasma CRP poorly correlates with disease severity in PUUV infection compared to plasma IL-6 [12]. This finding was consolidated by the present study, where the rise in CRP did not associate with severe AKI and the ROC curves didn’t show any diagnostic ability of CRP to find severe AKI. In the ROC curves maximum plasma resistin level had the best diagnostic accurancy to indicate severe AKI compared to leukocytes, IL-6 and CRP.

Many markers of inflammation are altered during acute PUUV infection, but not much is known about their relationship with proteinuria, which in turn seems to have a clear association with disease severity [18]. Increased capillary leakage seems to contribute to the amount of proteinuria during acute PUUV infection [18, 45], and some inflammation markers may have a more pronounced effect on this than others. Resistin has been previously related to vascular permeability and endothelial activation. In emergency department patients with sepsis, resistin and NGAL correlated with the expression of the endothelial cell adhesion molecules (VCAM-1, ICAM-1) [42] and were associated with septic shock, but not with mortality [41]. Any possible association with AKI was not discussed.

Plasma levels of adiponectin did not show any association with maximum creatinine values or albuminuria in hospital-treated patients in the present study. Low plasma adiponectin had, however, negative correlation to inflammatory markers CRP and IL-6. Adiponectin is a multifunctional cytokine with mainly anti-inflammatory properties [20]. Among adipokines, adiponectin is the most abundant in human serum. Elevated adiponectin concentrations are seen in many inflammatory diseases, such as rheumatoid arthritis, systemic lupus erythematosus, and inflammatory bowel disease, as well as in chronic kidney disease (CKD) [20]. Adiponectin has functions protective against obesity-related diseases but its concentration is decreased in obesity. The view of a possible role of adiponectin in AKI is mainly based on animal studies [46]. A protective effect of adiponectin has been shown in a murine model of ischemia-reperfusion -induced AKI [47]. If adiponectin has any protective effect on the development of AKI in humans, remains unclear.

Leptin deficient mice are susceptible to severe AKI caused by lipopolysaccharide induced endotoxic shock [48]. In the present study, leptin levels were decreased at the acute phase of PUUV infection, but even after adjustment by BMI, low leptin level did not correlate with the severity of AKI. When plasma leptin levels were compared between CKD patients on hemodialysis, patients with AKI, and healthy subjects, the patients with AKI were shown to have similar leptin levels as healthy subjects, while the level was clearly elevated in hemodialysis patients [49]. After kidney transplantation, the level of plasma leptin is shown to decline already during the first day [50]. Although kidneys are considered as the main site of leptin metabolism, the alterations of leptin level are possibly influenced by many coexisting factors affecting both its synthesis and degradation [49]. Circulating leptin level is highly correlated with fat mass and leptin concentrations have been reported to decrease in healthy subjects following fasting [51]. Anorexia, nausea and abdominal pain, and associated reduced food intake, frequently reported symptoms in NE, could partly explain low acute-phase leptin levels in the present study. It can’t be excluded, however, that low leptin at the acute phase of PUUV infection may contribute to the development of AKI.

There are scarce previous data about adipokine levels in patients with AKI. It is possible that the rise in plasma resistin level reflects accumulation of that particular adipokine during reduced glomerular filtration rate (GFR), since plasma resistin concentrations have been found to be elevated in uremia [52]. The molecular weight of resistin is relatively low 12,5 kDa compared to leptin 16 kDa and albumin 66 kDa. The molecular weight of adiponectin different multimers are 180–360 kDa. We believe that decreased GFR but also an increased synthesis of resistin can influence the elevation of plasma resistin level. In the present study, resistin had strong association with proteinuria and hematuria. In the multivariate analysis high resistin level remained (along with male gender) as an independent risk factor while the high amount of dipstick albuminuria (3+) did not. To summarize, the observed rise in plasma resistin level may not be explained by mere accumulation, but also increased synthesis, and thus resistin might have an independent influence on the development of AKI. As a limitation, we were not able to determine the point of time after the onset of fever when resistin peaks, because of varying delay of patient admission. The time course between resistin peak and the most severe AKI phase remains to be investigated.

In conclusion, plasma resistin concentration is elevated in acute PUUV hantavirus infection and it declines early during the recovery phase. Plasma resistin level is related to the severity of AKI, as well as to the amount of proteinuria and hematuria. An increased plasma resistin concentration was found to be an independent risk factor for severe AKI in PUUV infected patients also when adjusted to dipstick albuminuria. Further studies are needed to understand the role of resistin as an AKI biomarker or possible treatment target in patients with PUUV infection.

## Supporting information

S1 Dataset The complete data used in the analysis is in the excel file appendix. (XLSX)
